# Relationship Between C-Reactive Protein and Physical Fitness, Physical Activity, Obesity and Selected Cardiovascular Risk Factors in Schoolchildren

**Published:** 2010

**Authors:** Hamid Reza Sadeghipour, Ameneh Rahnama, Mohsen Salesi, Nader Rahnama, Hossein Mojtahedi

**Affiliations:** 1 Department of Exercise Sciences, Kazeroun University of Payam Nour, Kazeroun, Iran; 2 Education and Training Organization, Darab, Iran; 3 Department of Exercise Sciences, Shiraz University, Shiraz, Iran; 4 Professor of Sport Medicine, Department of Nutrition, Food and Exercise Sciences, Florida State University, Tallahassee, USA; and Department of Exercise Sciences, University of Isfahan, Isfahan, Iran; 5 Assistant Professor of Exercise Physiology; Faculty of Physical Education and Sports Sciences, University of Isfahan, Isfahan, Iran

**Keywords:** Cardiovascular diseases, C-reactive protein, Fitness, Body mass index

## Abstract

**Objectives:**

The aim of this study was to investigate the relation between C-reactive protein (CRP) with physical fitness, physical activity, obesity, and selected cardiovascular risk factors in school-children.

**Methods:**

Forty-four boy schoolchildren (mean ± SD: age 10.25 ± 0.75 years, height 144 ± 0.2 cm, body weight 46.1 5± 4.59 kg, body mass index 22.16 ± 2.16 kg/m^2^) voluntarily participated in this study. Physical fitness and physical activity were assessed using the 20-meter fitness test. Adiposity was estimated using body mass index. Blood samples were taken after an overnight fast and measured for CRP, LDL, HDL and cholesterol. Pearson’s correlation was calculated to determine the relations between these factors.

**Results:**

Mean (SD) CRP concentration was 1.07 (0.82) mg/l. A significant correlation was observed between CRP and VO2max (r=-0.45, P= 0.001), body mass index (r=0.55, P=0.000) and cholesterol (r=-0.35, P=0.04). No significant relation was found between CRP and physical activity, LDL and HDL (P> 0.05). Moreover, significant associations were observed between body mass index and VO2max (r=-0.33, P=0.02) and physical activity (r=-0.43, P=0.04).

**Conclusions:**

Body mass index was the most powerful predictor of serum concentrations of CRP in schoolchildren. It may be an important factor to control body weight to prevent an increase in serum CRP in children and to help the primordial prevention of chronic diseases.

## INTRODUCTION

Cardiovascular diseases (CVD) are the financial burden on the health system which continues to be one of the leading causes of morbidity and mortality especially in most developed countries.^1^,^2^ It is well-established that CVD is the first cause of death throughout the Western world and the second most common cause worldwide.^3^ Although atherosclerotic process typically occurs most frequently during or after the fifth decade of life,^4^ it is widely accepted that the atherosclerotic process begins in childhood and progresses through adulthood.^5^ Ribeiro and co-worker (2004) noted that 50% of children have almost one or more inflammatory CVD risk factors.^6^ The most important risk factors for development of CVD in children include low physical activity, obesity, high ratio of LDL to HDL and family history of heart disease.^1^

Moreover, investigations implicated that the main systemic inflammatory factors in atherosclerosis and cardiovascular disease include fibrinogen, interleukin-6 (IL-6), and C-reactive protein (CRP).^2^,^7^,^8^ CRP is an acute phase reactant usually associated with serious infection and inflammation, hence increase in CRP is the sign of presence of inflammatory disorders.^9^ It is recognized as a powerful predictor of cardiovascular risk factors, also type 1 diabetes and metabolic syndrome in healthy individuals and patients with known coronary artery disease.^7^–^11^ Results showed that elevation of CRP levels within the normal range is associated with an increased risk of atherosclerotic coronary heart diseases.^12^ For example, Yoshida et al. studied the relationship between serum CRP and other cardiovascular risk factors and reported that children with high CRP concentrations had higher body mass index (BMI), insulin resistance, uric acid, adipocytokines, LDL, apolipo-protein A-I, interleukin-6 (IL-6), and tumour necrosis factor (TNF).^13^ Although Pankow et al. (2001) expressed that genetic has the main role on CRP concentration,^14^ previous studies ascertain that obesity is a strong predictor of CRP concentration.^15^,^16^ Also, physical activity showed inversely correlated with CRP levels in adults.^17^–^20^ Although cardiovascular risk factor begins in childhood, data regarding inflammatory factors in children are not so well-developed as in adults. Recently, Thomas et al. (2008) reported a positive significant relationship between CRP and obesity in children but no significant relationship showed with physical fitness.^2^ Isasi and coworker (2003) reported that physical fitness is inversely correlated with CRP levels in children.^21^ The Committee on Atherosclerosis, Hypertension and Obesity in Youth (AHOY) recently issued a statement concerned with cardiovascular health promotion for children and emphasized that schools were important stake-holders in population-based health promotion and risk-reduction efforts.^22^ Because there are relatively few studies in children, therefore the aim of this study was to investigate CRP concentration in school children and identify the relationship between CRP with obesity, physical activity and physical fitness.

## MATERIAL AND METHODS

Forty-four primary boy students from Shiraz schools volunteered to participate in this study (mean ± SD: age 10.25 ± 0.75 years, height 144 ± 0.2 cm, body weight 46.15 ± 4.59 kg, BMI 22.16 ± 2.16 kg/m^2^). Informed consent form was obtained from the parents of all children. This study was approved by the University of Payame Nour Fars. Body mass was recorded using an electronic scale and the barefoot stature was measured by tape meter. Then, body mass index (BMI) was calculated as the mass divided by stature squared (kg/m^2^) for each subject. It should be noted that BMI is the most frequently used clinical indicator of overweight in young people and adults. Obesity estimated as BMI >95^th^ percentile.^1^,^2^,^23^ Physical fitness level was assessed by using 20-metre shuttle run test which is one of the best predictors of maximal aerobic fitness especially in young people. This test involved continuous running between two lines of 20 meters apart. Each subject stood behind the line and began running when instructed by the recorded voice on CD or audiotape. The subjects were told to keep up with the pacer until exhausted. The subjects’ scores were the number of shuttles performed before exhaustion.^2^ For measurement of CRP (mg/l), LDL (mg/dl), HDL (mg/dl) and cholesterol (mmo/l), blood obtained in the morning after an overnight fast, between 8 and 10 am. CRP was measured with high sensitivity enzyme-linked immunosorbent assay (hs-ELISA), and the values were expressed in milligrams per liter (mg/l). Also, routine chemical methods used to determine the serum concentrations of total cholesterol (TC), HDL-C and LDL-C. In this study, total daily physical activity was assessed using 7-day recall questionnaire.^24^ All children were asked to recall their activities over the previous seven days and record in the questionnaire. In this questionnaire, some activities were defined. For each activity, subjects were asked to report how often, how long and how intense took place their exercise bouts. SPSS software was used for analysis of data and the level of significance set at P<0.05. Quantitative variables were demonstrated as mean ± standard deviation (SD). Pearson’s correlations were calculated to determine the relations between CRP and physical fitness, physical activity level, adiposity and other cardiovascular risk factors.

## RESULTS

Table 1 presents the selected characteristics of subjects and Table 2 shows the Pearson correlations between variables. Mean C-reactive protein concentration was 1.07 mg/L. A significant correlation was observed between CRP and VO2max (r=− 0.45, P=0.001), body mass index (r=0.55, P<0.0001) and cholesterol (r=−0.35, P=0.04). No significant relation was found between CRP and physical activity, LDL-C and HDL-C. Moreover, significant associations were observed between body mass index and VO2max (r=−0.33, P=0.02) and physical activity (r=−0.43, P=0.04).

**Table 1:** The characteristics the study variables

Variables	Mean	SD
CRP (mg/L)	1.07	0.82
Body mass index (kg/m^2^)	22.16	2.16
VO_2max_	52.30	2.25
Physical activity (minutes)	83.12	11.4
LDL-C (mg/dL)	109.63	32.16
HDL-C (mg/dL)	63.91	21.68
Cholesterol (mg/dL)	172.85	42.15

**Table 2:** Pearson’s correlation coefficient (r) of C-reactive protein with selected factors

	VO_2max_	BMI	LDL-C	HDL-C	TC	Physical activity
CRP(mg/L)	-0.045	0.55	0.17	-0.22	0.35	-0.31
	0.001*	<0.0001*	0.25	0.13	0.04*	0.08

BMI: body mass index

TC: total cholesterol

* P<0.05

## DISCUSSION

The purpose of this study was to investigate the relation between CRP with physical fitness, physical activity, obesity, and selected cardiovascular risk factors in schoolchildren. In this study, the mean CRP was 1.07 mg/l, but in 12 children, the level of CRP was higher than the healthy level (>3 mg/L).^2^ Some researchers noted that the high concentration of CRP effected CVD risk in later life but it was not proved completely, hence large cohort size studies and longitudinal studies are necessary to ascertain whether raised CRP concentrations during childhood and adolescence lead to an increased risk of CVD in later life or not. In our study, there was a negative significant correlation between CRP levels and physical fitness. This finding is in agreement with those reported by Isasi and co-workers and Hamer,^19^,^21^ but in the study of Thomas et al. although there was a negative correlation between these two factors, this correlation wasn’t significant.^2^ Although studies of physical fitness and CRP are limited and more studies are needed, this negative correlation demonstrated that high physical fitness directly affects cardiovascular system and consequently decreases the risk of coronary diseases.^17^ Thomas and co-workers noted that the 20-metre shuttle run test is accepted as a valid and reliable test of aerobic fitness in young people; hence, we presumed that the levels achieved were good estimates and the results were reliable. Results showed no significant correlation between CRP and physical activity which was in accordance with previous studies.^1^,^2^ However, our measures of physical activity involved self-reported questionnaires and subjects may report wrong time and duration of their physical activity. Also, it is possible that when CRP is in the normal range, exercise and daily physical activity have little effect.^2^,^16^,^17^ However, there is evidence that physical activity may modify the inflammatory process and decrease the heart disease risk^17^,^24^ but the mechanism through which physical activity could be associated with lower levels of inflammation markers is unknown. It seems that our subjects’ physical activity levels, both inside and outside of schools, are low and this can increase the risk of cardiovascular diseases. Hence, higher levels of physical activity can lower the concentrations of four out of five inflammation markers such as CRP.^17^ In our study, there was a positive significant correlation between CRP with BMI and hence obesity. This finding was in agreement with some previous studies.^2^,^17^,^24^ It seems that with increase of body mass index, obesity develops and CRP level elevates. The mechanisms responsible for the association between obesity and CRP are not yet clearly understood. It is possible that adipose tissue is a direct source of pro-inflammatory cytokines such as TNF-a and IL-6, which in turn act as stimuli for CRP synthesis in the liver.^2^,^25^ Also, in our study similar with other studies,^8^,^17^ physical activity was significantly associated with decreased body mass index. These results showed that physical activity in leisure time can decrease body mass index and obesity, therefore can reduce the cardiovascular risk factors, and may reduce CRP levels adequately by reducing adiposity.^18^ Results showed a significant relationship between CRP and cholesterol but not between CRP and LDL or HDL-C. Cook et al. and Ford reported that serum concentrations of CRP were associated with HDL, but not with other lipid parameters.^24^,^26^ Yoshida et al. reported a relationship between CRP and HDL-C, LDL-C and total cholesterol.^13^ High number of subjects in previous studies can be one of the differences, and future studies with numerous subjects are needed to investigate whether this relation exists in young people.

In conclusion, an association between CRP and selected cardiovascular risk factors such as 20-meter shuttles, BMI and cholesterol were found in this study. Most of previous studies reported that BMI was the most powerful predictor of serum concentrations of CRP in schoolchildren. These findings suggest that it may be important to control body weight to prevent an increase in serum CRP in children.
